# Network analysis reveals potential mechanisms that determine the cellular identity of keratinocytes and corneal epithelial cells through the Hox/Gtl2-Dio3 miRNA axis

**DOI:** 10.3389/fcell.2025.1475334

**Published:** 2025-01-17

**Authors:** Yanjie Guo, Weini Wu, Haoyu Chen, Xueqi Wang, Yi Zhang, Shuaipeng Li, Xueyi Yang

**Affiliations:** Life Science College, Luoyang Normal University, Luoyang, Henan, China

**Keywords:** keratinocytes, corneal epithelial cells, cellular identity, Hox genes, Gtl2-Dio3 miRNAs

## Abstract

During embryonic development, both corneal epithelial cells (CECs) and keratinocytes (KCs) originate from the surface ectoderm. As a result of this shared origin, corneal epithelial cells may exhibit the same characteristics as the skin epidermis in pathological situations, while keratinocytes are ideal seed cells for tissue-engineered corneas. However, how the identities of keratinocytes and corneal epithelial cells are determined is currently unclear. In this study, to investigate the molecular mechanisms determining the identity of keratinocytes and corneal epithelial cells, small RNA and mRNA sequencing analyses of these two cell types were performed. Analysis of the sequencing data revealed that almost all the miRNAs in the Gtl2-Dio3 imprinting region were highly expressed in keratinocytes and accounted for 30% of all differentially expressed miRNAs (DEMs). Since all the genes in the Gtl2-Dio3 imprinting region form a long polycistronic RNA under the control of the Gtl2 promoter, we next examined the expression of transcription factors and their binding near the Gtl2 locus. The findings indicated that the homeobox family dominated the differentially expressed transcription factors, and almost all *Hox* genes were silenced in corneal epithelial cells. Transcription binding site prediction and ChIP-seq revealed the binding of Hox proteins near the Gtl2 locus. Analysis of the Gtl-Dio3 miRNA target genes indicated that these miRNAs mainly regulate the Wnt signaling pathway and the PI3K-Akt signaling pathway. The crucial transcription factors in corneal epithelial cells, *Pax6*, *Otx2*, and *Foxc1*, are also targets of Gtl-Dio3 miRNAs. Our study revealed potential mechanisms that determine the cellular identity of keratinocytes and corneal epithelial cells through the Hox/Gtl2-Dio3 miRNA axis, which provides a new perspective for understanding the developmental regulation of corneal epithelial cells and the mechanisms of corneal opacity, as well as for establishing the groundwork for promoting the transdifferentiation of keratinocytes into corneal epithelial cells.

## 1 Introduction

Keratinocytes and corneal epithelium are both composed of stratified squamous epithelial cells and share surface ectoderm as a common origin in development (Gonzalez et al., 2018). During development, stem cells in the basal layer of embryonic skin give rise to keratinocyte stem cells, which are the common origin of all keratin-expressing cells, including keratinocytes and corneal epithelial cells (Irfan-Maqsood, 2013). Therefore, these cells share many similarities in function, homeostasis maintenance and molecular markers (Nowell and Radtke, 2017). Nonetheless, their cell fates are essentially different, ultimately resulting in a transparent corneal epithelium and an opaque epidermis. However, the mechanisms that regulate the fate of keratinocytes and corneal epithelial cells are poorly understood. The phenomenon of mutual transdifferentiation between skin epidermal cells and corneal epithelial cells provided us with some inspiration. Under pathological conditions, corneal epithelial cells may exhibit characteristics of skin epidermal cells, such as the expression of epidermis-specific keratin, downregulation of the cornea-specific transcription factor PAX6, and epithelial vascularization, resulting in corneal opacity (Nowell and Radtke, 2017; Ouyang et al., 2014). Loss of *PAX6* expression or *PAX6* interference in limbal stem cells similarly triggers the transition in the corneal epithelium (Li et al., 2015; 2008; Ouyang et al., 2014), while retransfection of *PAX6* can partially restore the properties of the corneal epithelium (Li et al., 2008). In addition, corneal epithelial cells can undergo transdifferentiation to the skin epidermis in response to dermal signals, during which the expression level of *Pax6* gradually decreases and ultimately disappears (Pearton et al., 2005). Conversely, skin epidermal stem cells overexpressing *PAX6* can transdifferentiate into corneal limbal stem cells that, when transplanted, can successfully repair corneal defects on the ocular surface, forming a transparent corneal epithelial cell layer expressing keratin 3 and 12 (Ouyang et al., 2014). Recent studies have indicated that RUNX1 and SMAD3 can interact with PAX6 to form a core transcription regulatory circuitry (CRC) that cooperatively controls superenhancers to maintain the properties and homeostasis of the corneal epithelium (Li et al., 2021). These findings highlight the regulatory network that maintains corneal epithelial homeostasis, with Pax6 at its core. The key difference between keratinocytes and corneal epithelial cells, whether in embryos or adults, lies in the expression of *Pax6* (Gehring and Ikeo, 1999). Pax6, an early eye field transcription factor, plays a role in the development of corneal epithelial cells, and both the lens and corneal epithelium are affected by the expression of Pax6 in the surface ectoderm (Lwigale, 2015). However, surface ectoderm that does not express Pax6 forms skin epidermis (Leiper et al., 2009; Li et al., 2008). In addition to Pax6, the transcription factors Otx and Foxc1 also play critical roles in the regulation of corneal development. The Otx genes are essential for tissue specification in the developing eye (Martinez-Morales et al., 2001), and eye field transcription factors such as Pax6, Six3, Rx, or Six6 are induced by Otx2 (Zuber et al., 2003). Mutations in *Otx2* can cause the loss of the cornea, iris, and lens (Matsuo et al., 1995). Aberrant expression of the *Otx* genes has been detected in a variety of ocular pathologies (Azzolini et al., 2013; Ragge et al., 2005), and the *Otx* gene is involved in the repair or regeneration processes of ocular pathologies (Azzolini et al., 2021; Mitashov, 2007). The transcription factor Foxc1 is able to regulate corneal transparency through vascular endothelial growth factor (VEGF) signaling and is known to be co-expressed with Pax6 in corneal epithelial cells (Koo and Kume, 2013). The depletion of *Foxc1* has been implicated in the transformation of corneal epithelial cells into skin-like epithelial cells (Li et al., 2021).

The Wnt signaling pathway also significantly regulates the homeostasis of corneal epithelial cells. As a key signaling pathway in dermal condensation, Wnt signaling determines the differentiation of the basal layer of the embryonic skin into the skin and its appendages (Zhang et al., 2009) and regulates the functions of different skin cells (Lim and Nusse, 2013). Under the control of dermal signals, corneal epithelial cells undergo transdifferentiation to the skin epidermis, during which the expression level of β-catenin is sharply upregulated (Pearton et al., 2005), indicating that the Wnt signaling pathway is activated. Knocking out the Wnt signaling pathway inhibitory gene *Dkk2* in corneal epithelial cells also leads to transdifferentiation into skin epidermis and the formation of skin appendages such as hair follicles (Mukhopadhyay et al., 2006). Moreover, a complex regulatory association exists between the Wnt/β-catenin signaling pathway and Pax6. The activation of Wnt/β-catenin signaling inhibits *Pax6*, resulting in the loss of cornea-specific gene expression (Fuhrmann, 2008; Mukhopadhyay et al., 2006). *Pax6* can induce the expression of Wnt/β-catenin inhibitors such as *sFRP*, *Dkk1*, and *Wif1*, thus maintaining the inhibitory state of Wnt/β-catenin signaling in the corneal epithelium (Davis and Piatigorsky, 2011). However, the molecular mechanisms regulating the identity of keratinocytes and corneal epithelial cells remain poorly understood. Therefore, comparing the molecular characteristics of keratinocytes and corneal epithelial cells may reveal the factors underlying the differences in cell identity and enhance the understanding of the regulatory mechanisms governing corneal epithelial cell development and diseases of corneal opacity. These comparisons can also facilitate the transdifferentiation of keratinocytes into corneal epithelial cells.

In our study, the differences in mRNA and miRNA expression between keratinocytes and corneal epithelial cells were evaluated. We identified specific transcription factors in keratinocytes and corneal epithelial cells and found that *Hox* genes, which were silenced in corneal epithelial cells, may act as upstream controllers by regulating miRNA expression in the Gtl2-Dio3 imprinted region and determining the molecular identity of keratinocytes and corneal epithelial cells.

## 2 Materials and methods

### 2.1 Cell isolation and culture

Mouse keratinocytes were isolated as described previously (Li et al., 2017). The skin was removed from the back of 1–3°days old mice and cut into 0.5 × 0.5 cm pieces. The skin was transferred, epidermal side up, into a 35 mm dish containing 2 mL of Dispase II. The dish was placed in 4°C to digest overnight. The following day, the mouse skin was retrieved and the epidermis was separated, and it was washed 3-4 times in PBS. The epidermis was transferred into a new culture dish and further cut into 0.1 × 0.1 cm pieces with scissors. Trypsin (0.25%) was added to the culture dish and it was digested at 37°C for 10–15 min. Complete culture medium (Procell, Wuhan, China) was added to terminate the digestion process and the cells were centrifuged at 1,000 rpm for 5 min. The supernatant was discarded and the cells were resuspended in complete culture medium. The cells were seeded in new dishes and then transferred to an incubator for culture, with the medium changed every 2–3°days.

Mouse corneal epithelial cells were isolated as described previously (Kobayashi et al., 2009). After the mouse had been euthanized by cervical dislocation, the eyeballs were removed using tweezers and soaked in sterile PBS for 5 min. A circular incision was made around the limbus, the cornea was flipped so that the epithelial side was facing up, and it was placed into a sterile culture dish. It was digested overnight at 4°C with Dispase II. The loosened corneal epithelial sheets were gently peeled off with tweezers and incubated in 0.25% trypsin at 37°C for 10 min. Complete culture medium (Procell, Wuhan, China) was added to terminate the digestion process and the cells were centrifuged at 1,000 rpm for 5 min. The supernatant was discarded and the cells were resuspended in complete culture medium. The cells were seeded in new dishes and then transferred to an incubator for culture, with the medium changed every 2–3°days.

All animal experiments were conducted in accordance with the guidelines and approval of the Animal Care and Welfare Committee of Luoyang Normal University (Approval code is 0020080A).

### 2.2 mRNA and sRNA sequencing

As described previously (Guo et al., 2023), in the second passage of primary culture, when the cell confluence exceeds 80%, total RNA of keratinocytes and corneal epithelial cells (Three biological replicates for each cell sample) was extracted using TRIzol Reagent (Thermo Fisher Scientific, DE, USA) according to the manufacturer’s instructions. Then the analysis on 1.5% agarose gels revealed RNA degradation and possible DNA contamination. The determination of RNA concentration and purity was carried out utilizing the NanoDrop 2000 Spectrophotometer (Thermo Fisher Scientific, DE, USA). Additionally, the assessment of RNA integrity was performed using the RNA Nano 6000 Assay Kit within the Agilent Bioanalyzer 2,100 System (Agilent Technologies, CA, USA).

The mRNA sequencing was performed essentially as described in detail previously (Guo et al., 2023), a total of 1.5 μg RNA per sample was utilized as the input material for removing rRNA with the Ribo-Zero rRNA Removal Kit (Epicentre, Madison, WI, USA). To prepare sequencing libraries, the NEBNextR UltraTM Directional RNA Library Prep Kit for IlluminaR (NEB, MA, USA) was employed, following the recommendations of the manufacturer, and index codes were incorporated to attribute sequences to each sample. In brief, Fragmentation was carried out by employing divalent cations at elevated temperature in NEBNext First Strand Synthesis Reaction Buffer (5X). The first strand cDNA was synthesized using a random hexamer primer and Reverse Transcriptase. Subsequently, the second-strand cDNA synthesis was carried out using DNA Polymerase I and RNase H. The remaining overhangs were converted into blunt ends through exonuclease/polymerase activities. Following the adenylation of 3′ ends of DNA fragments, NEBNext Adaptor with a hairpin loop structure was ligated for hybridization preparation. To preferentially select insert fragments of 150–200 bp in length, the library fragments underwent purification with AMPure XP Beads (Beckman Coulter, Beverly, USA). Subsequently, 3 μL of USER Enzyme (NEB, MA, USA) was utilized with size-selected, adaptor-ligated cDNA at 37°C for 15 min before conducting PCR. The PCR was carried out using Phusion High-Fidelity DNA polymerase, Universal PCR primers, and Index(X) Primer. Finally, the PCR products were purified using the AMPure XP system, and the library quality was assessed using the Agilent Bioanalyzer 2,100 and qPCR. After the library quality check, different libraries were pooled based on the target sequencing data output and then sequenced using the Illumina platform (NovaSeq 6,000).

The sRNA sequencing was performed essentially as described in detail previously (Guo et al., 2023), a total of 2.5 ng of RNA per sample served as the input material for the RNA sample preparations. The sequencing libraries were prepared using the NEBNextR UltraTM small RNA Sample Library Prep Kit for IlluminaR (NEB, USA), following the recommendations of the manufacturer, and index codes were incorporated to attribute sequences to each sample. Initially, the 3′ SR Adaptor was ligated. A mixture of 3′ SR Adaptor for Illumina, RNA, and Nuclease-Free Water was incubated for 2 min at 70° in a preheated thermal cycler, then transferred to ice. Subsequently, 3′ Ligation Reaction Buffer (2X) and 3′ Ligation Enzyme Mix were added to ligate the 3′ SR Adaptor, followed by an incubation for 1 h at 25°C in a thermal cycler. To prevent adaptor-dimer formation, the SR RT Primer hybridized to the excess 3′ SR Adaptor and transformed the single-stranded DNA adaptor into a double-stranded DNA molecule. The second step involved ligating the 5′ SR Adaptor, followed by reverse transcription to synthetic the first chain. Finally, PCR amplification and size selection were carried out. Fragment screening was performed using PAGE gel electrophoresis, and the fragments were recycled by rubber cutting to obtain small RNA libraries. Subsequently, the PCR products were purified using the AMPure XP system, and the library quality was evaluated using the Agilent Bioanalyzer 2,100 system. After the library quality check, different libraries were pooled based on the target sequencing data output and then sequenced using the Illumina platform (NovaSeq 6,000).

### 2.3 Comparative analysis

For mRNA sequencing, the adapter sequences and low-quality sequences were eliminated from the datasets, thereby converting the raw reads into clean reads. The clean reads were then compared with the reference genome sequences and further analyzed and annotated for the completely matched reads. The HISAT2 software was employed for mapping the clean reads with the reference genome.

For sRNA sequencing, the Bowtie software tools were used to filter rRNA, tRNA, snRNA, snoRNA, other non-coding RNA, and repeats. Filtered reads were employed for identifying known miRNAs and predicting novel miRNAs by comparing them with the miRBase database. For the prediction of novel miRNA secondary structures, the Randfold software tool was utilized.

### 2.4 Differential expression analysis

Differential expression analysis was performed essentially as described in detail previously using the DESeq R package (Guo et al., 2023). The resulting P-values were adjusted using the Benjamini and Hochberg approach to control the false discovery rate. Genes identified by DESeq with an adjusted P-value≤0.01 and an absolute |log2(Fold change)| ≥1 were designated as differentially expressed.

### 2.5 Functional enrichment and network construction

As described previously (Guo et al., 2023), the Gene Ontology (GO) Enrichment Analysis of differentially expressed genes (DEGs) was carried out using the clusterProfiler R package, applying hypergeometric testing to identify significantly enriched GO entries in comparison to the entire genome background. Pathway analysis was performed using the KEGG database (http://www.genome.jp/kegg/). The clusterProfiler R package was employed to identify significantly enriched KEGG pathways relative to the entire genome background. The DEG sequences were blasted (blastx) against the genome of a related species with available protein–protein interaction (PPI) data on the STRING database (http://string-db.org/) to acquire the predicted PPI of the DEGs.

### 2.6 Data visualization

Ribbon plot and correlation analysis of Gtl2-Dio3 miRNAs expression, Sankey diagram of KEGG pathway were carried out through Hiplot Pro (https://hiplot.com.cn/). The DNase-seq, ATAC-seq data and ChIP-seq data for Hox, RNA polymerase II (Pol II) were downloaded from ChIP-Atlas (https://chip-atlas.org/) and then visualized using the WashU Epigenome Browser (http://epigenomegateway.wustl.edu/browser/).

### 2.7 miRNA targets and luciferase reporter assay

The miRNA target genes supported by CLIP-seq data were downloaded from StarBase (https://rnasysu.com/encori/). The wild type (WT) and mutation (MUT) 3′UTRs of predicted target genes were cloned into the luciferase reporter plasmid psi-CHECK2 for the luciferase reporter assay. Then, WT or MUT plasmids were co-transfected with 100 ng miRNA mimics or miR-NC (Sangon, Shanghai, China) into HEK293T cells using Lipo6000TM (Beyotime, Jiangsu, China) for 48 h. The Dual Luciferase Reporter Gene Assay Kit (Beyotime, Jiangsu, China) was employed to measure the activity of luciferase according to the provided guidelines. The firefly luciferase values were normalized using the ratio of firefly to Renilla luciferase.

### 2.8 Statistical analysis

For statistical analysis, all numerical results are expressed as the mean ± standard error of the mean (SEM) derived from a minimum of three separate experiments. We employed Student’s t-test to assess statistically significant differences between the two data sets, with P-values ≤0.05 being regarded as indicative of statistical significance.

## 3 Results

### 3.1 miRNAs in the Gtl2-Dio3 imprinted region were differentially expressed between keratinocytes and corneal epithelial cells

**FIGURE 1:**
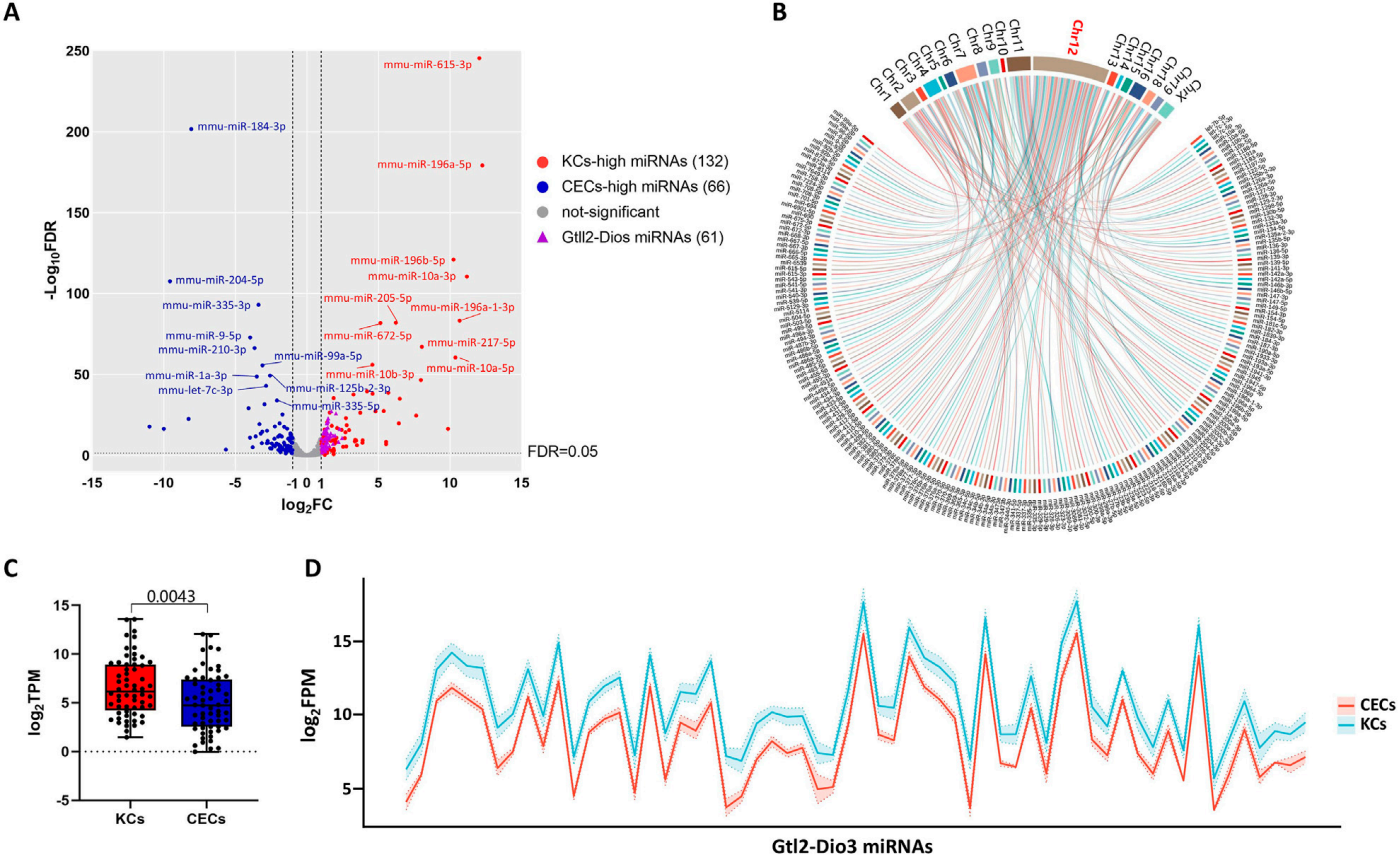
miRNAs in the Gtl2-Dio3 imprinted region were differentially expressed in keratinocytes and corneal epithelial cells. **(A)** Volcano plot for miRNA pairwise comparisons of keratinocytes and corneal epithelial cells. **(B)** Distribution of differentially expressed miRNAs (DEMs) on chromosomes. **(C)** Box plot for DEMs in Gtl2-Dio3 imprinted region and **(D)** Ribbon plot for all miRNAs in Gtl2-Dio3 imprinted region showing the Gtl2-Dio3 miRNAs in corneal epithelial cells exhibit global downregulation. For all underlying data, see PRJNA1197694 in NCBI.

To explore the molecular mechanisms that determine the identities of keratinocytes (KCs) and corneal epithelial cells (CECs), we used primary cells isolated from mouse skin and cornea. The two types of cultured cells exhibited characteristic keratin markers of keratinocytes (Krt5) and corneal epithelial cells (Krt12), and were able to stably proliferate *in vitro* (Supplementary Figure S1). Then we first performed small RNA sequencing (sRNA-seq) of keratinocytes and corneal epithelial cells in the second passage of primary culture (the data accession number is PRJNA1197694 in NCBI). The overall statistics of the mRNA sequencing data are presented in Supplementary Table S1. Through pairwise comparison, we identified 198 differentially expressed miRNAs (DEMs) between KCs and CECs with a |log2 (fold change)|≥1 and adjusted P value (Padj)≤0.01 as the criteria (Figure 1A). Out of the DEMs, 132 were found to have increased expression in KCs, which we designate as KCs-high miRNAs. Conversely, 66 DEMs exhibited higher expression levels in CECs, which we label as CECs-high miRNAs (Figure 1A).

Subsequently, to investigate the potential roles of these DEMs, we analyzed the distribution of all 198 DEMs on the chromosomes. Impressively, we found that 63 DEMs (32%) were located on chromosome 12 (Figure 1B), and of these, 62 DEMs mapped to a miRNA cluster in 12qF1, known as the Gtl2-Dio3 imprinted region. Interestingly, all 62 miRNAs located in the Gtl2-Dio3 imprinted region (referred to as Gtl2-Dio3 miRNAs) are KCs-high miRNAs (Figure 1A). To ascertain that this is not a coincidental phenomenon, we attempted to investigate the global expression pattern of the Gtl2-Dio3 miRNAs. As shown in Figures 1B, C, miRNAs across the Gtl2-Dio3 locus exhibited global downregulation in corneal epithelial cells compared to keratinocytes, although a few miRNAs did not reach significance at Padj ≤0.01. These findings indicate that the collective expression of Gtl2-Dio3 miRNAs varies between keratinocytes and corneal epithelial cells.

**FIGURE 2:**
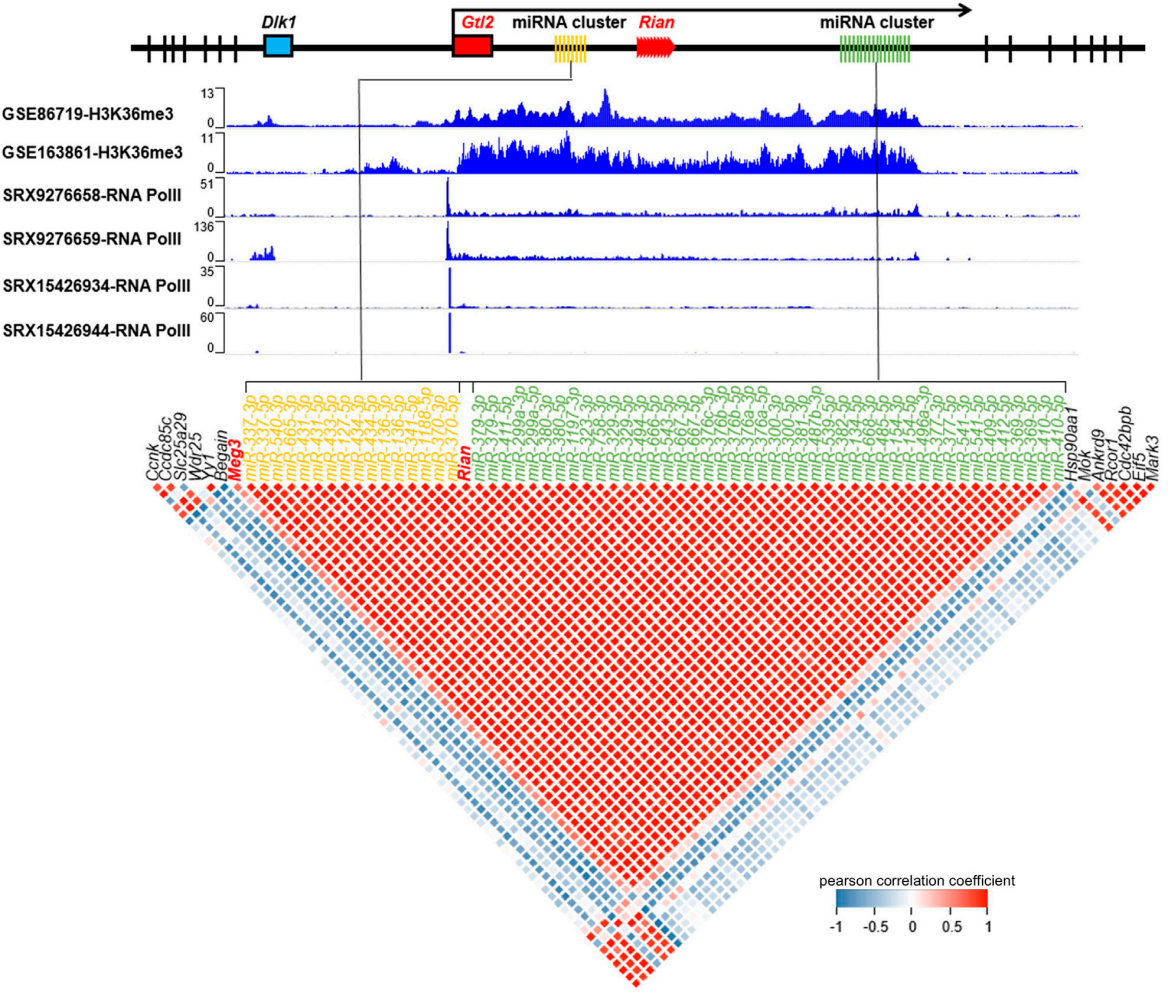
Gtl2-Dio3 expressed as a whole polycistronic transcript. The H3K36me3 and RNA pol II enrichment status in the Gtl2 locus and expression correlation of the Gtl2-Dio3 non-coding RNAs and flanking mRNAs show Gtl2-Dio3 expressed as a whole polycistronic transcript. RNA pol II exhibits binding specificity to the upstream region of the Gtl2-Dio3 locus in keratinocytes (SRX15426934 and SRX15426944) and dermal fibroblasts (SRX9276658 and SRX9276659), with no detectable binding in other regions. H3K36me3 ChIP-seq (GSE163861 for keratinocytes, GSE86719 for forebrain in which Gtl2-Dio3 locus is highly expressed) binding profiles, which are markers of transcription elongation, demonstrate continuous transcription of the Gtl2-Dio3 polycistron. A correlation plot illustrates the relationship between the expression of Gtl2-Dio3 miRNAs and flanking mRNAs in keratinocytes. Red coloration denotes a robust positive correlation between the Gtl2-Dio3 miRNAs clusters, MEG3 and Rian, while blue signifies a robust negative correlation. The miRNAs are arranged in the plot according to their location in the genome, with different colors used to distinguish different miRNA clusters within the Gtl2-Dio3 imprinted region.

Gtl2-Dio3 is understood to be expressed as a whole polycistronic transcript, which could account for the observed overall differences in the expression of Gtl2-Dio3 miRNAs between keratinocytes and corneal epithelial cells. To confirm this, we investigated the enrichment of RNA Pol II and H3K36me3 within the actively transcribed Gtl2-Dio3 region. To confirm the universality of whole polycistronic transcription of Gtl2-Dio3, in addition to keratinocytes (SRX15426934 and SRX15426944 for RNA Pol II, GSE163861 for H3K36me3), we included dermal fibroblasts (SRX9276658 and SRX9276659 for RNA Pol II) and forebrain (GSE86719 for H3K36me3) in our analysis, where the Gtl2-Dio3 region is actively expressed (Jacobs, 2019; Buechler et al., 2021). As shown in Figure 2, RNA pol II bound upstream of the Gtl2 locus in all keratinocytes and dermal fibroblasts, with the binding region encompassing the core promoter element, the TATA (TATATAAA) box (Supplementary File S1). In contrast, other parts of the Gtl2-Dio3 imprinted region showed no binding of RNA pol II. H3K36me3 typically exhibits enrichment within the gene body of actively transcribed genes (Bannister et al., 2005; Nojima et al., 2018; Wagner and Carpenter, 2012). The enrichment of H3K36me3 in both keratinocytes and forebrain samples showed that the H3K36me3 signal covers the entire Gtl2-Dio3 imprinting region (Figure 2). Moreover, we analyzed the coexpression of noncoding RNA in the Gtl2-Dio3 imprinted region and demonstrated a strong positive correlation between miRNA and the long noncoding RNAs within this region (*Meg3* and *Rain*) but not between the flanking genes at both ends of this region (Figure 2). The above results demonstrate that the Gtl2-Dio3 imprinted region produces polycistronic transcripts through the action of the Gtl2 upstream promoter. This results in a consistent expression pattern for the Gtl2-Dio3 miRNAs, with an overall downregulation in corneal epithelial cells compared to keratinocytes.

### 3.2 *Hox* genes were silenced in corneal epithelial cells

**FIGURE 3:**
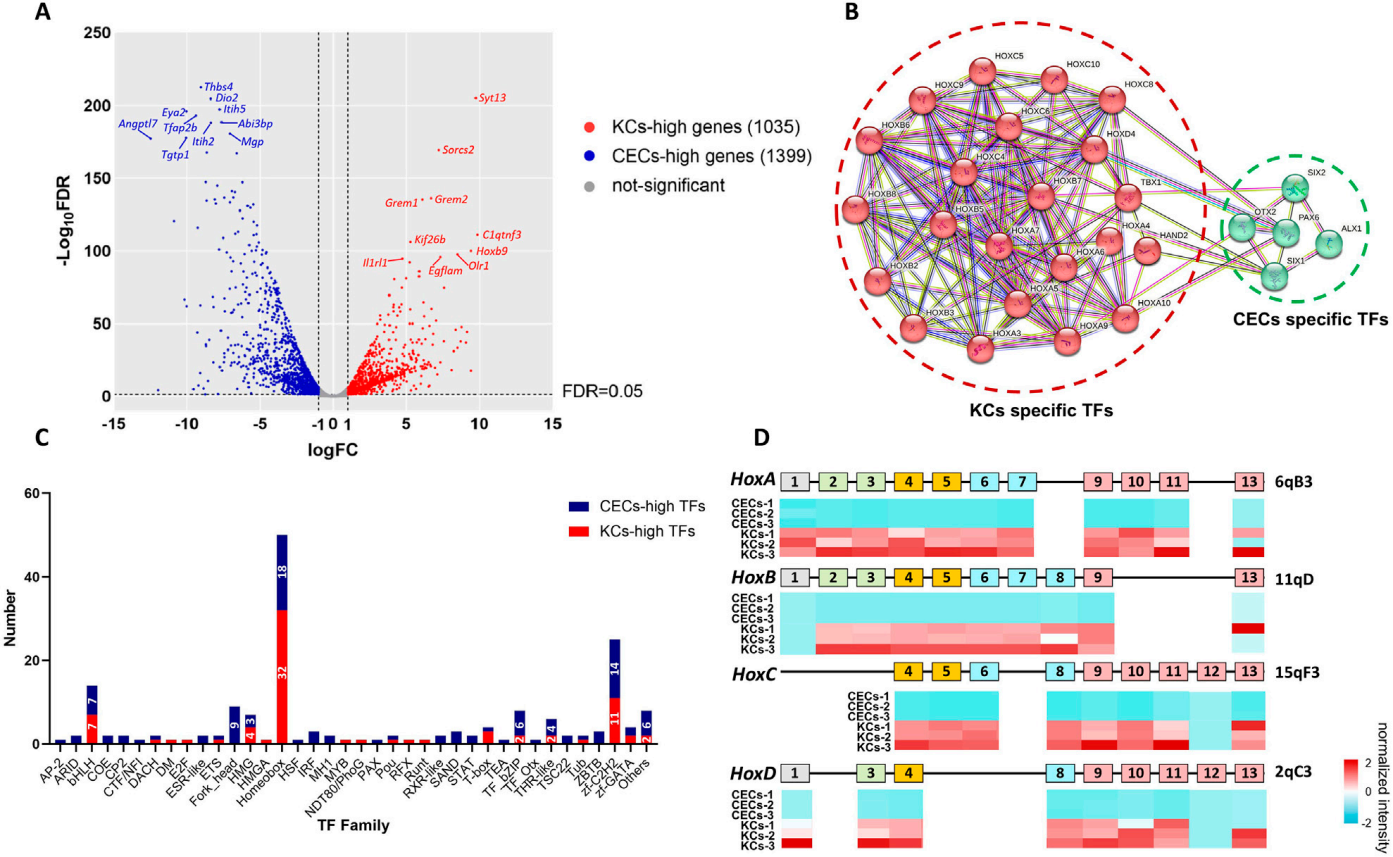
Identification of specific transcription factors (TFs) and Hox genes in keratinocytes and corneal epithelial cells. **(A)** Volcano plot for mRNA pairwise comparisons of keratinocytes and corneal epithelial cells. **(B)** PPI network for specific TFs of keratinocytes and corneal epithelial cells. Keratinocytes specific TFs include Hoxa1, Hoxa10, Hoxa3, Hoxa4, Hoxa5, Hoxa6, Hoxa7, Hoxa9, Hoxb2, Hoxb3, Hoxb5, Hoxb6, Hoxb7, Hoxb8, Hoxc10, Hoxc4, Hoxc5, Hoxc6, Hoxc8, Hoxc9, Hoxd4, Hand2 and Tbx1. corneal epithelial cells specific TFs include Pax6, Otx2, Six1, Six2 and Alx1. **(C)** The classification of all differentially expressed TFs. Among the TFs, 50 of 181 belonged to the homeobox family and the vast majority of which were Hox proteins. **(D)** Heatmap of Hox gene expression in keratinocytes and corneal epithelial cells show nearly all the Hox genes are silenced in corneal epithelial cells. For all underlying data, see PRJNA1197694 in NCBI.

To investigate the possible reasons for the differential expression of miRNAs in the Gtl2-Dio3 imprinted region, we performed mRNA sequencing analysis on both keratinocytes and corneal epithelial cells (the data accession number is PRJNA1197694 in NCBI). The overall statistics of the mRNA sequencing data are presented in Supplementary Table S2. Through pairwise comparison, we identified 2,434 differentially expressed genes (DEGs) between KCs and CECs with a |log2 (fold change)|≥1 and adjusted P value (Padj)≤0.01 as the criteria (Figure 3A). Out of the DEGs, 1,035 were found to have increased expression in KCs, which we designate as KCs-high genes. Conversely, 1,399 DEGs exhibited higher expression levels in CECs, which we label as CECs-high genes (Figure 3A). Among the CECs-high genes, we observed the inclusion of ocular field transcription factors such as Pax6, Otx2, and Six2 (Zuber et al., 2003), as well as factors that maintain corneal epithelial cells like Foxc1 and Pitx2 (Evans and Gage, 2005; Li et al., 2021). Notably, Pax6, Otx2, and Six2 are specifically expressed in corneal epithelial cells and not in keratinocytes. Among the KCs-high genes, we observed the inclusion of keratins Krt6 and Krt17, which are specific to keratinocytes, as well as Krt5 and Krt14, markers of the basal layer. The distinct expression patterns of these genes suggest that keratinocytes and corneal epithelial cells preserve their specific cellular identities *in vitro*.

To identify potential regulatory factors of Gtl2-Dio3 miRNAs, we analyzed the transcription factors among all 2,434 DEGs. A total of 181 transcription factors were included in these 2,434 DEGs. Among these transcription factors, five were specifically expressed in corneal epithelial cells (CEC-specific TFs, for which the FPKM value in keratinocytes was 0 in at least two of the three replicates): Pax6, Otx2, Six1, Six2 and Alx1 (Figure 3B; Supplementary File S2). Additionally, 23 transcription factors were specifically expressed in keratinocytes (KC-specific TFs). They are Hoxa1, Hoxa10, Hoxa3, Hoxa4, Hoxa5, Hoxa6, Hoxa7, Hoxa9, Hoxb2, Hoxb3, Hoxb5, Hoxb6, Hoxb7, Hoxb8, Hoxc10, Hoxc4, Hoxc5, Hoxc6, Hoxc8, Hoxc9, Hoxd4, Hand2 and Tbx1 (Figure 3B; Supplementary File S2). Among KC-specific TFs, Hand2 and Tbx1 are the major transcription factors that regulate vascularization (Cioffi et al., 2014; Dai and Cserjesi, 2002; Morikawa and Cserjesi, 2004; Vitelli et al., 2002), which is the main cause of corneal opacity. This could also be one of the reasons why the skin epidermis appears as an opaque stratified epithelium. Surprisingly, the remaining 21 transcription factors were all found to be Hox proteins (Figure 3B; Supplementary File S2). Subsequently, we carried out a classification of all 181 transcription factors. The results indicated that homeobox family proteins constituted a significant proportion, specifically 50 out of 181 (Figure 3C; Supplementary File S2). Among these, the vast majority were Hox proteins, amounting to 26 out of 50 (Figure 3C; Supplementary File S2). Vertebrates have four *Hox* gene clusters, referred to as *Hox* loci, which are designated *Hoxa*, *Hoxb*, *Hoxc*, and *Hoxd* in mice (Shah and Sukumar, 2010). These 4 *Hox* gene clusters contained 13 paralogous groups with a total of 39 genes (Pascual-Anaya et al., 2013). Examination of the expression of all 39 *Hox* genes in keratinocytes and corneal epithelial cells revealed that all the *Hox* genes except for *Hoxb1*, *b13*, *c12*, and *d12* were expressed in keratinocytes, while nearly all the *Hox* genes were silenced in corneal epithelial cells (Figure 3D; Supplementary File S3). In our study, the expression patterns of *Hox* genes in keratinocytes and corneal epithelial cells were consistent with findings from other studies (Hayashi et al., 2016; Smits et al., 2023). This collectively indicates that, despite a common origin from the surface ectoderm, the development of corneal epithelium does not require *Hox* gene regulation compared to skin epidermis. Particularly, the results from Smits and colleagues (Smits et al., 2023) showed that *Hox* genes are specific to keratinocytes compared to limbal stem cells. Combining our results, we believe that the silencing of *Hox* genes does not occur during the differentiation of limbal stem cells into corneal epithelial cells, but rather during the development of the surface ectoderm into the corneal epithelium. In summary, *Hox* genes specifically expressed in keratinocytes may play a role in determining the identity of keratinocytes and corneal epithelial cells.

### 3.3 Direct binding of Hox proteins to the Gtl2 locus

**FIGURE 4:**
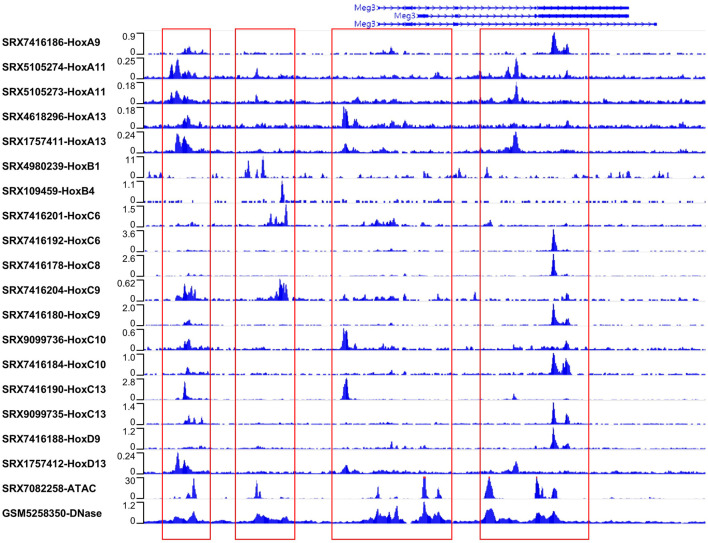
Hox proteins directly binding to the Gtl2 locus. ATAC-seq, DNase-seq and Hox binding signals in the Gtl2 locus. ATAC-seq (SRX7082258 for keratinocytes) and DNase-seq (GSM5258350 for forebrain) reveal four chromatin accessible regions near Meg3. The four chromatin accessible regions (chr12:1,09518669-109521055, chr12:109527173-109531218, chr12:109538996-109548641 and chr12:109552685-109562123) were marked with red box. All available Hox ChIP-seq datasets (the accession number are shown) in the ChIP-Atlas database are download to analyze binding at the Gtl2 locus. Shown are ChIP-seq binding profiles of all Hox proteins at the four chromatin accessible regions.

As transcription factors, Hox proteins can regulate the expression activity of target genes by binding to their upstream regions (Carnesecchi et al., 2018). To investigate whether *Hox* genes might regulate miRNA expression in the Gtl2-Dio3 imprinted region by binding to the Gtl2 locus, we initially employed ATAC-seq and DNase-seq data to evaluate the chromatin accessibility of the Gtl2-Dio3 imprinting region in the active state. Similarly, to confirm that the chromatin accessibility state is consistent in the actively expressed Gtl2-Dio3 imprinted region across different samples, we analyzed the ATAC-seq data from keratinocytes (SRX7082258) and the DNase-seq data from forebrain (GSM5258350) (Figure 4). As shown in Figure 4, in both keratinocytes and forebrain, there are four chromatin accessible regions near Meg3 that roughly span the following regions on the chromosome: chr12:1,09518669-109521055, chr12:109527173-109531218, chr12:109538996-109548641 and chr12:109552685-109562123. After obtaining the DNA sequences of these four regions, we used the JASPAR database to predict the binding situations of Hox proteins in these four regions. The results showed that almost all the existing Hox proteins in the database had potential binding sites in these four regions (Supplementary File S4).

Then, we attempted to obtain all available ChIP-seq datasets for Hox proteins from the public database ChIP-Atlas to validate the prediction results. As shown in Figure 4, among the 18 ChIP-seq datasets that included 12 Hox proteins, the following were identified: SRX7416186 (HoxA9), SRX109459 (HoxB4), SRX7416201 (HoxC6), SRX7416192 (HoxC6), SRX7416178 (HoxC8), SRX7416204 (HoxC9), SRX7416180 (HoxC9), SRX9099736 (HoxC10), SRX7416184 (HoxC10), SRX7416190 (HoxC13), SRX9099735 (HoxC13), and SRX7416188 (HoxD9), originated from ES-derived embryoid bodies (EBs); SRX5105274 (HoxA11) and SRX5105273 (HoxA11), SRX4618296 (HoxA13), SRX1757411 (HoxA13), and SRX1757412 (HoxD13), derived from embryonic forelimb buds; and SRX4980239 (HoxB1), derived from embryonic stem cells (ESCs). All of the Hox proteins show binding signals at the Gtl2 locus. Comparing the chromatin regions with these binding signals to the chromatin accessible regions reveals a high degree of consistency, and the binding positions align with the predicted locations (Figure 4; Supplementary File S4). These findings suggest that Hox proteins may regulate miRNA expression in the Gtl2-Dio3 imprinted region by binding directly to the Gtl2 locus.

### 3.4 The Wnt and PI3K-Akt signaling pathways play a central role in determining the identity of keratinocytes and corneal epithelial cells

**FIGURE 5:**
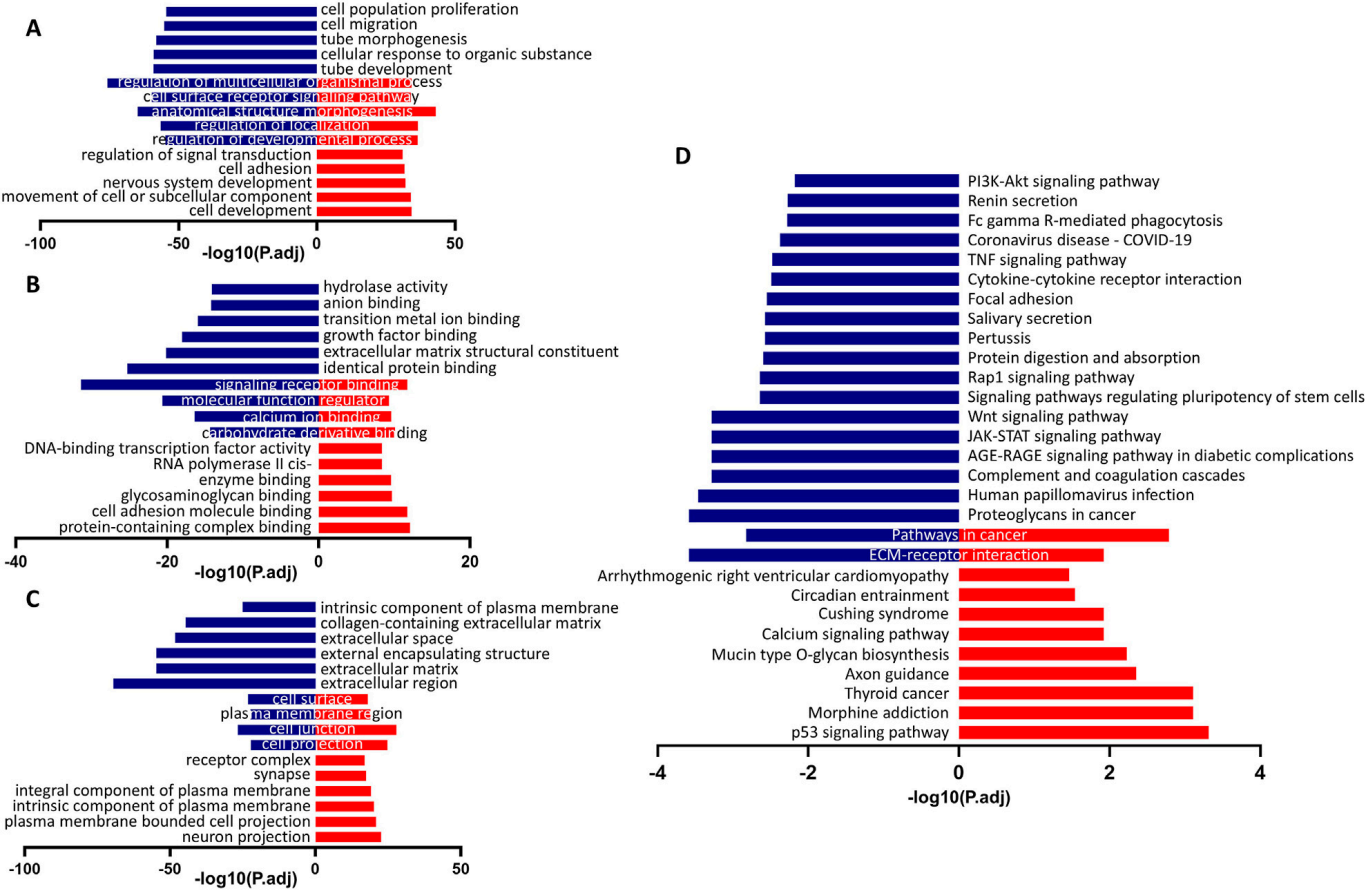
The GO and KEGG pathway analysis of differentially expressed genes (DEGs) in keratinocytes and corneal epithelial cells. **(A)** The top 10 enriched biological process (BP) terms. **(B)** The top 10 enriched cellular component (CC) terms. **(C)** The top 10 enriched molecular function (MF) terms. **(D)** The top 20 enriched KEGG pathways. Blue columns were indicated the terms with CECs-high genes, and red columns were indicated the terms with KCs-high genes. For all underlying data, see PRJNA1197694 in NCBI.

We next sought to elucidate the downstream events regulated by Gtl2-Dio3 miRNAs by network analysis of the mRNA and sRNA-seq data. GO and KEGG pathway analyses were subsequently conducted on the KCs-high and CECs-high genes (Figure 5). The top 10 enriched biological process (BP), cellular component (CC) and molecular function (MF) terms are presented in Figures 5A–C. The GO terms enriched in KCs-high genes are primarily related to “cell adhesion”, while the GO terms enriched in CECs-high genes are mainly associated with “cell migration” and “extracellular matrix”, such as “cell migration”, “tube morphogenesis”, “tube development”, “extracellular matrix structural constituent”, “collagen-containing extracellular matrix”, “extracellular space”, “external encapsulating structure”, “extracellular matrix”, and “extracellular region”. This may correspond to the different states of movement, proliferation, and differentiation of keratinocytes and corneal epithelial cells. The KEGG pathway analysis revealed that the pathways enriched for KCs-high genes mainly include the “p53 signaling pathway” and “Calcium signaling pathway”, while the pathways enriched for CECs-high genes mainly include the“PI3K-Akt signaling pathway”, “TNF signaling pathway”, “Focal adhesion”, “Rap1 signaling pathway”, “Wnt signaling pathway” and “JAK-STAT signaling pathway” (Figure 5D), suggesting that these signaling pathways play different roles in keratinocytes and corneal epithelial cells.

**FIGURE 6:**
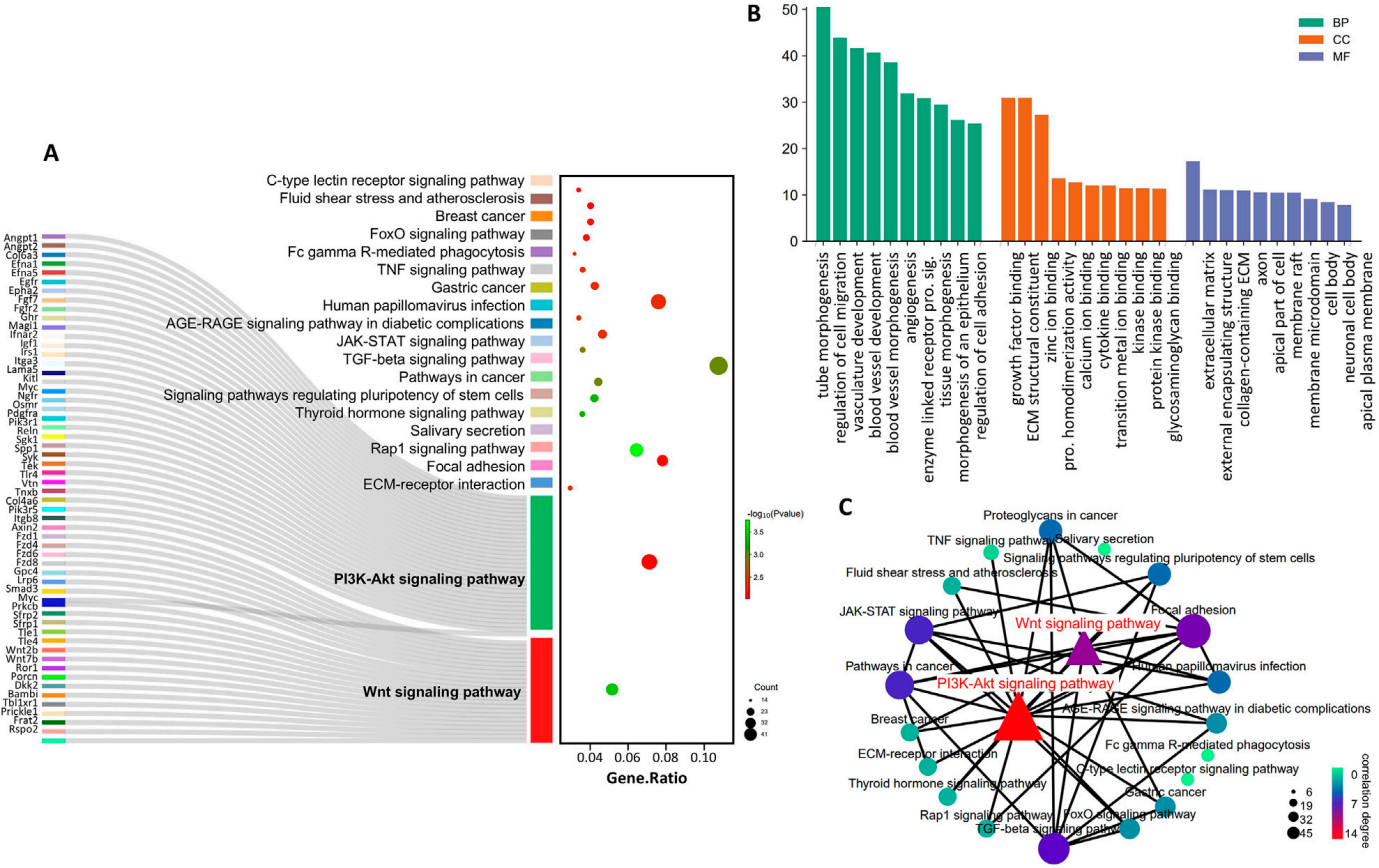
Identification of the Gtl2-Dio3 miRNAs target hub signaling pathways. **(A)** The sankey diagram of top 20 KEGG pathway enriched by intersection genes. The genes in the Wnt and PI3K-Akt signaling pathways are all displayed. **(B)** The top 10 BP, CC and MF terms enriched by intersection genes. **(C)** The pathway network of the top 20 KEGG pathways. The Wnt signaling pathway and the PI3K-Akt signaling pathway are identified as hub signaling pathways.

Subsequently, we obtained target genes supported by CLIP-seq data from the starBase database and determined the intersection of the Gtl2-Dio3 miRNAs targets with the downregulated DEGs in KCs compared to CECs (CECs-high genes), based on the understanding that miRNAs can downregulate the expression of target genes. GO pathway analysis of these intersection genes revealed that the commonly enriched GO terms of the intersection genes and the downregulated DEGs included “collagen-containing extracellular matrix”, “extracellular space”, “extracellular matrix”, “extracellular region” and “extracellular matrix structural constituent” (Figure 6B), suggesting that Gtl2-Dio3 miRNAs target the “extracellular matrix” to influence the characteristics of keratinocytes and corneal epithelial cells. KEGG pathway analysis of intersection genes revealed that the commonly enriched KEGG pathways included “ECM-receptor interaction”, “Wnt signaling pathway”, “focal adhesion”, “PI3K-Akt signaling pathway”, “Fc gamma R-mediated phagocytosis”, “TNF signaling pathway”, “JAK-STAT signaling pathway”, “signaling pathways regulating pluripotency of stem cells”, “salivary secretion” and “Rap1 signaling pathway”. These pathways showed a high overlap with the KEGG enrichment results of downregulated DEGs (Figure 6A). Among these pathways, the Wnt signaling pathway and the PI3K-Akt signaling pathway were identified as hub signaling pathways (Figure 6C). The above results indicate that miRNAs within the Gtl2-Dio3 imprinted region are principal factors contributing to the differential gene expression observed in keratinocytes and corneal epithelial cells. Furthermore, the Wnt and PI3K-Akt signaling pathways appear to be the predominant mechanisms by which the Gtl2-Dio3 miRNAs mediate their effects.

### 3.5 Gtl2-Dio3 miRNAs target directly to Wnt and PI3K-Akt signaling pathways and key transcription factors of CECs

**FIGURE 7:**
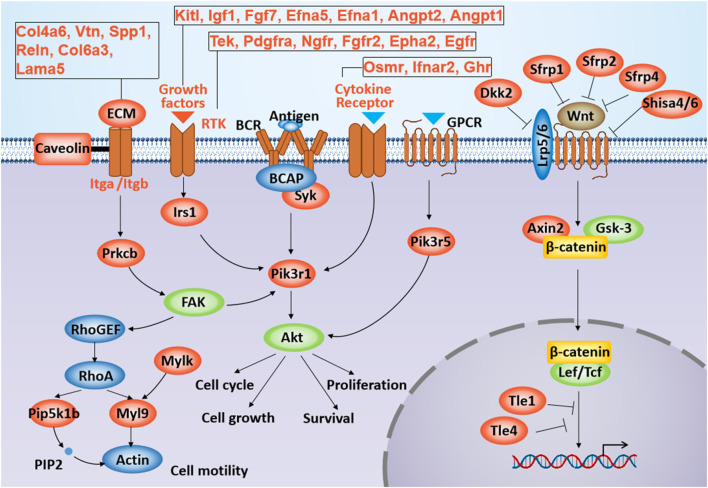
The location and role of Gtl2-Dio3 target genes in the Wnt and PI3K-Akt signaling pathway. Shown is schematic representation of the Wnt and PI3K-Akt signaling pathways. The Gtl2-Dio3 target genes in the Wnt and PI3K-Akt signaling pathway were labeled in red.

We next sought to investigate whether the Gtl2-Dio3 miRNAs could directly regulate the Wnt and PI3K-Akt signaling pathways. Clustered miRNAs typically act in a similar manner on the same downstream target genes or multiple functionally related target genes (Kim and Nam, 2006; Yu et al., 2006). For this reason, we analyzed the target genes of Gtl2-Dio3 miRNAs within the hub signaling pathway, and the results revealed that multiple genes within both the Wnt and PI3K-Akt signaling pathways are targeted by Gtl2-Dio3 miRNAs (Figure 7). Furthermore, we investigated the distribution and function of these target genes within their respective signaling pathways. Interestingly, we discovered that Gtl2-Dio3 miRNAs exert distinct regulatory effects on the Wnt and PI3K-Akt signaling pathways. In the PI3K-Akt signaling pathway, target genes of Gtl2-Dio3 miRNAs are distributed throughout the entire pathway, playing a crucial role in the activation of the PI3K-Akt signaling pathway (Figure 7). This suggests that miRNAs from the Gtl2-Dio3 imprinted region exert an inhibitory effect on the PI3K-Akt signaling pathway. In contrast, within the Wnt signaling pathway, target genes of Gtl2-Dio3 miRNAs cover almost all inhibitory genes of the entire pathway (Figure 7), indicating that Gtl2-Dio3 miRNAs can relieve the inhibition of these genes on the Wnt signaling pathway, thereby activating the Wnt signaling pathway. More interestingly, when comparing the changes in Wnt and PI3K-Akt signaling pathways during skin and corneal development, we observed that PI3K-Akt signaling activity progressively diminishes throughout the development of the skin epidermis, with a significant drop postnatally to levels that are nearly undetectable (O’Shaughnessy et al., 2007). An elevation in PI3K-Akt signaling activity is associated with functional disruptions in the skin epidermis (Elkenani et al., 2016). Conversely, the activation of the Wnt signaling pathway is an initial promoter of skin development, and the activation of the Wnt signaling pathway in corneal epithelial cells is accompanied by the transdifferentiation into skin epidermal cells. The above results are highly consistent with the role of Gtl2-Dio3 miRNAs in the Wnt and PI3K-Akt signaling pathways in our study, suggesting that their differential expression is pivotal in determining the cellular identities of keratinocytes and corneal epithelial cells.

**FIGURE 8:**
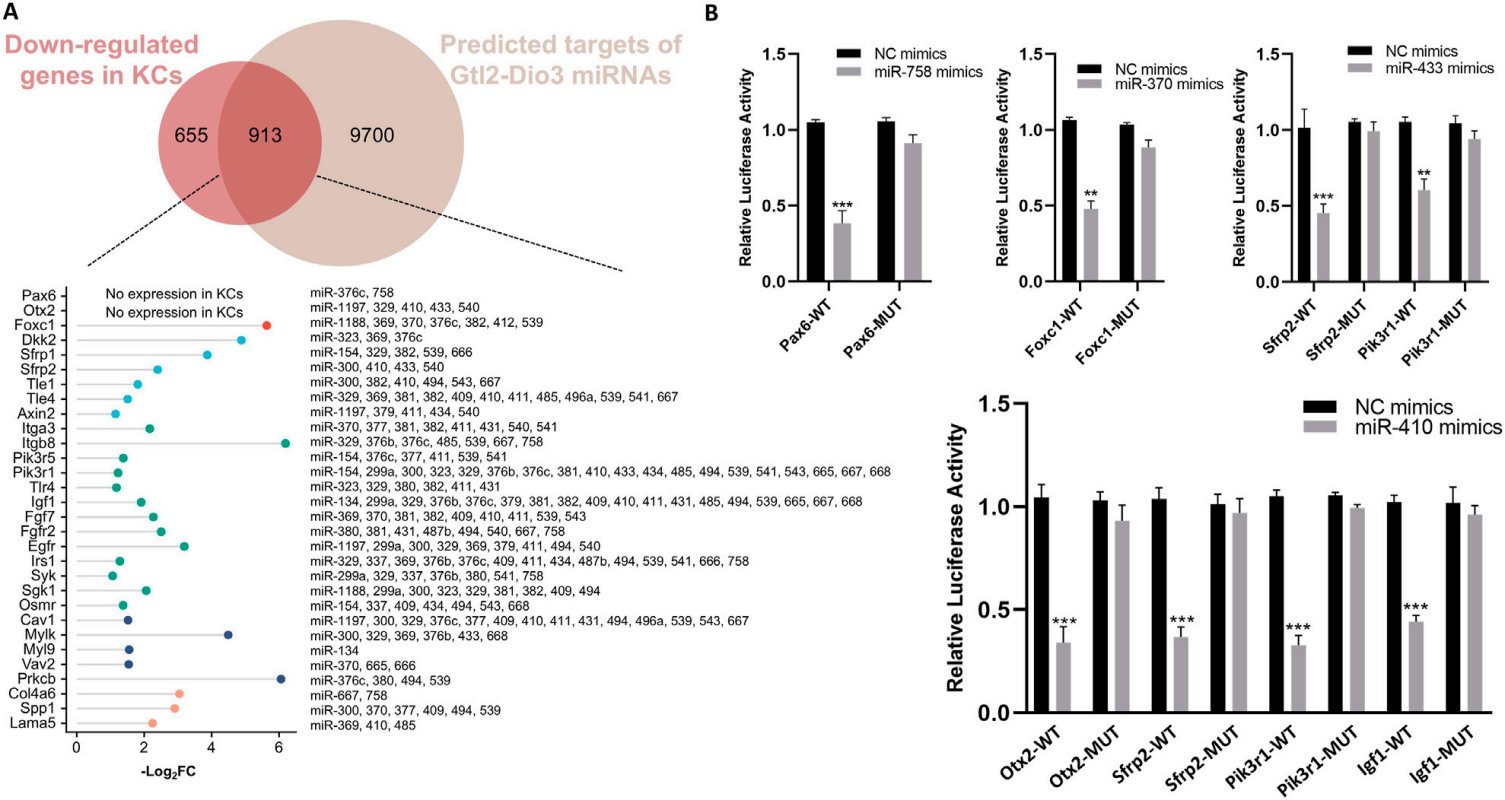
Identification the target genes of the Gtl2-Dio3 miRNAs. **(A)** The putative Gtl2-Dio3 miRNAs and their corresponding target genes. The 3′UTRs of the intersecting genes in the Wnt and PI3K-Akt signaling pathways are analyzed, with all putative Gtl2-Dio3 miRNAs displayed subsequent to their respective target genes. **(B)** Luciferase reporter assays performed to validate the target genes of Gtl2-Dio3 miRNAs. *P < 0.05, **P < 0.01, ***P < 0.001.

Furthermore, we analyzed the 3′UTRs of Gtl2-Dio3 miRNAs target genes within the hub signaling pathway. The results demonstrated that these genes contain one or multiple conserved binding sites of Gtl2-derived miRNAs in their respective 3′UTRs (Figure 8A). Unexpectedly, in addition to the genes associated with the Wnt and PI3K-Akt signaling pathways, we also found that Pax6, Otx2 and Foxc1, which are the key regulatory factors of corneal epithelial cells, are also the target genes of Gtl2-Dio3 miRNA (Figure 8A). This further supports the role of Gtl2-Dio3 miRNAs in determining the fate of keratinocytes and corneal epithelial cells. Subsequently, dual-luciferase reporter assays were utilized to determine whether these genes were direct targets of Gtl2-Dio3 miRNAs. The WT and MUT 3′UTRs of selected genes (*Pax6*, *Otx2*, *Foxc1*, *Sfrp2*, *Igf1*, and *PiK3r1*) were cloned and inserted into the luciferase reporter plasmid psi-CHECK2. The recombinant plasmids and the corresponding miRNA mimics or nonspecific control (NC) mimics were then transfected into HEK293T cells. The results showed that miR-758 directly targeted *Pax6*, miR-410 targeted *Otx2* and *Igf1*, miR-370 targeted *Foxc1*, and miR-433 and 410 targeted *Sfrp2* and *Pik3r1* (Figure 8B). The above results indicated that Gtl2-Dio3 miRNAs determine the identity of keratinocytes and corneal epithelial cells through the regulation of the Wnt, PI3K-Akt signaling pathway, and key transcription factors. Considering the regulatory role of *Hox* genes on Gtl2-Dio3 miRNA, we believe that the Hox/Gtl2-Dio3 miRNA axis is the key to determining the fate of keratinocytes and corneal epithelial cells. During the development of the surface ectoderm, *Hox* genes maintain the developmental environment of the skin epidermis through Gtl2-Dio3 miRNA. However, the silencing of *Hox* genes leads to the withdrawal of the signals related to the development of the skin epidermis, which in turn triggers the developmental process towards the corneal epithelium.

## 4 Discussion

**FIGURE 9:**
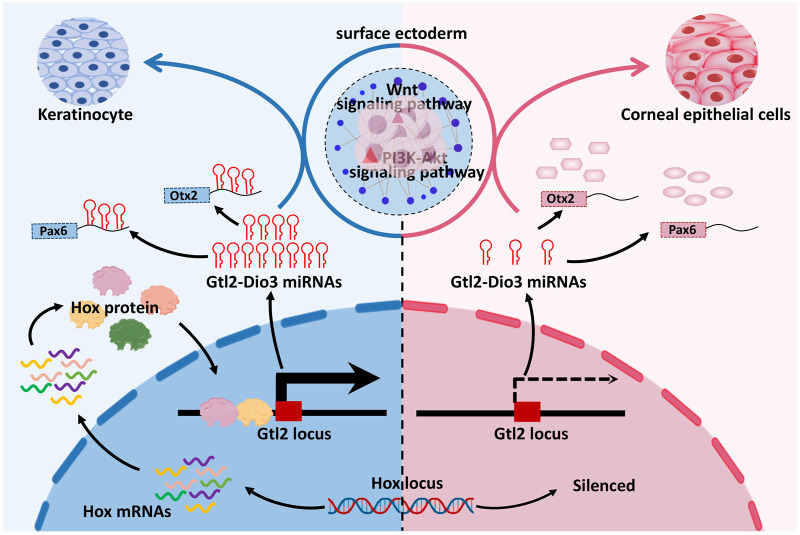
Mechanisms of cellular identity determination in keratinocytes and corneal epithelial cells. *Hox* genes exhibited global silencing in corneal epithelial cells and were able to regulate miRNA expression in Gtl2-Dio3 imprinted region by binding to Gtl2 locus, and then Gtl2-Dio3 miRNAs could affect the identity of keratinocytes and corneal epithelial cells by regulating Wnt signaling pathway, PI3K-Akt signaling pathway and key transcription factors.

In this study, through network analysis of sRNAs and mRNAs in keratinocytes and corneal epithelial cells, we showed that *Hox* genes, key transcription factors involved in embryonic development and cell fate determination, exhibited global silencing in corneal epithelial cells. The expression levels of miRNAs in the Gtl2-Dio3 imprinted region in corneal epithelial cells showed an overall downregulation compared with keratinocytes. Further investigation revealed that *Hox* genes can directly bind to the Gtl2 locus and that Gtl2-Dio3 miRNAs subsequently target the Wnt and PI3K-Akt signaling pathways and key transcription factors of corneal epithelial cells (Figure 9). The Hox/Gtl2-Dio3 miRNA axis was identified as a potential mechanism that determines the cellular identity of keratinocytes and corneal epithelial cells.

### 4.1 The withdrawal of skin development-related signals in the surface ectoderm is an important trigger of corneal epithelial development

Both corneal epithelial cells and keratinocytes originate from the surface ectoderm during embryonic development (Gonzalez et al., 2018). Pax6, the key transcription factor for corneal development, is not expressed in the surface ectoderm but gradually emerges during development toward the cornea (Leiper et al., 2009; Li et al., 2008). Analysis of the existing evidence showed that in mice, *PAX6* expression was first detected in the surface ectoderm at the E8 stage and subsequently persisted in the developing corneal epithelium (Grindley et al., 1995; Li et al., 2015). Investigation of the causes of *Pax6* expression in chickens and mice revealed that corneal stromal signals fail to stimulate *Pax6* expression in uncommitted epidermal cells, even from the stroma in the limbal region (Collomb et al., 2013). The ablation results also indicated that corneal epithelial cells could still be produced in the absence of lenses. Furthermore, the formation of corneal epithelial cells remains unaffected by inhibition of the BMP signaling pathway, which is the final lens-promoting event, even before the scheduled separation of the lens from the surface ectoderm (Collomb et al., 2013). These results suggest that the expression of *Pax6* is not induced by signals from the corneal stroma or the lens and that the influencing factors may be derived from intrinsic changes in the surface ectoderm. A complex regulatory association exists between the Wnt/β-catenin signaling pathway and Pax6. Activation of Wnt/β-catenin signaling inhibits *Pax6*, resulting in the loss of cornea-specific gene expression (Fuhrmann, 2008; Mukhopadhyay et al., 2006). *Pax6* can induce the expression of Wnt/β-catenin inhibitors such as *sFRP*, *Dkk1*, and *Wif1*, thus maintaining the inhibitory state of Wnt/β-catenin signaling in the corneal epithelium (Davis and Piatigorsky, 2011). During the differentiation of induced pluripotent stem cells (iPSCs) into the ocular surface ectoderm cell lineage, ocular surface epithelium formation requires the inhibition of Wnt signaling, without which the cells would otherwise develop into the skin epidermis, and the expression of cellular endogenous *Dkk1* and *Sfrp2* could act as Wnt inhibitors to initiate ocular surface ectoderm development (Kobayashi et al., 2020). These results collectively indicate that Wnt/β-catenin signaling hinders corneal epithelial development while initially promoting skin development (Chang et al., 2004) and has high activity in the basal and spinous layers of the developing skin (Wang et al., 2020). Both the early developmental stage and the fully developed corneal epithelium are capable of differentiating into the epidermis after recombining with the dermis of the skin (Ferraris et al., 2000; Pearton et al., 2005). This differentiation process is accompanied by the rapid loss of *Pax6* and activation of the Wnt/β-catenin signaling pathway (Chang et al., 2004; Gat et al., 1998; Pearton et al., 2005; Zhang et al., 2008). The corneal stroma and lens were unable to induce the differentiation of the epidermis into the corneal epithelium, whereas the skin dermis could induce the differentiation of the corneal epithelium into the epidermis. The above evidence indicates that the corneal epithelium is affected by the withdrawal of skin development-related signals in the surface ectoderm, providing a permissive environment for the expression of corneal epithelium development-related transcription factors such as Pax6 and Otx2. This model is consistent with our results. Research has demonstrated the expression of *Hox* genes within the surface ectoderm (Taniguchi, 2014; Zhang et al., 2021), and comparisons of transcription factors in KCs and LSCs have similarly revealed that *Hox* genes are specifically expressed in KCs (Smits et al., 2023). This finding indicates that the silencing of *Hox* genes does not occur during differentiation from LSCs to CECs but rather during development from the surface ectoderm to the cornea. As in our results, the silencing of *Hox* genes during this process led to the downregulation of miRNA expression in the Gtl2-Dio3 imprinted region, thereby relieving the inhibition of Wnt inhibitory genes, causing the withdrawal of the Wnt signal, and resulting in the expression of corneal epithelium development-related transcription factors such as Pax6 and Otx2.

### 4.2 The expression and redundancy of the *Hox* genes in the skin and corneal epithelium

The homeobox family of transcription factors is defined by the presence of the homeobox sequence, which is a conserved 180 bp DNA region that enables Hox proteins to share a homologous domain of 60 amino acids as a DNA-binding domain capable of binding to TAAT motifs (Desplan et al., 1985; Gehring et al., 1994; Passner et al., 1999). These vertebrates share four *Hox* gene clusters, referred to as *Hox* loci, which are denoted as *Hoxa*, *b*, *c*, and *d* in mice (Shah and Sukumar, 2010). Within these 4 *Hox* gene clusters, there are 13 paralogous groups with a total of 39 genes (Pascual-Anaya et al., 2013). Different *Hox* genes exhibit distinct expression patterns in the skin. For example, strong expression of *HOXA4*, *HOXA5*, and *HOXA7* can be detected in fetal skin, whereas the expression of *HOXA6*, *HOXA10*, *HOXA11*, and *HOXB7* is comparatively weaker. In addition to *HOXA4*, *HOXA5*, and *HOXA7*, *HOXB1*, *HOXB3*, *HOXB6*, and *HOXC4* have been detected in newborn skin. *HOXB7* exhibits low expression in both the basal and suprabasal layers of 10-week embryonic skin, and by 21 weeks, it becomes widespread throughout the epidermis. In the skin of newborns and adults, the expression of *HOXB7*, *HOXC4*, and *HOXB4* is restricted to the upper epidermis (Stelnicki et al., 1998). In mice, the expression of *Hoxd9* and *Hoxd11* is limited to the caudal skin at 14.5 days of gestation. *Hoxd13* appears at 2 days after birth and is expressed exclusively in epidermal cells (Kanzler et al., 1994). Although comprehensive spatial and temporal expression studies of *Hox* genes during skin development have yet to be conducted, it is certain that *Hox* genes act as major regulatory genes in skin development (Scott and Goldsmith, 1993; Stelnicki et al., 1998), specifying the regional characteristics of the skin during morphogenesis according to their distinct expression patterns (Stelnicki et al., 1998; Yamaguchi et al., 2005). Although share a common origin, the development of the corneal epithelium does not seem to require regulation by *Hox* genes in the same way that skin epidermis development does. Several research findings suggest that *Hox* genes are not expressed in cells on the ocular surface (Mallo and Alonso, 2013; Pearson et al., 2005). The differentiation of iPSCs into the ocular ectodermal cell lineage also demonstrated that within the self-formed ectodermal autonomous multi-zone (SEAM) generated by iPSC differentiation, *Hox* gene-nonexpressing cells formed the corneal epithelium, whereas *Hox* gene-expressing cells primarily formed nonocular epithelial cells, with a small proportion contributing to the conjunctival epithelium (Hayashi et al., 2016). Our study, similar to a recent study, also demonstrated that *Hox* genes exhibit specificity for keratinocytes and are not expressed in corneal epithelial or limbal stem cells (Smits et al., 2023). Furthermore, in our study, all *Hox* genes except *Hoxb1*, *b13*, *c12*, and *d12* were expressed in keratinocytes. Among these genes, *Hoxa1*, *a2*, *a3*, *a4*, *a10*, *a11*, *a13*, *c11*, *c13*, *d1*, *d10*, *d11* and *d13* exhibited lower expression levels, whereas the remaining *Hox* genes had higher expression levels. During development, *Hox* genes typically function in clusters, including paralogous genes from different clusters as well as adjacent genes within the same cluster, to achieve target regulation (La Celle and Polakowska, 2001). The conservation of the homology domain determines the shared target genes among Hox transcription factors, which exhibit significant redundancy (Denans et al., 2015; Iacovino et al., 2009; Lacombe et al., 2013). Several results support the phenomenon of functional redundancy in Hox transcription factors, which specifically manifests in the downstream regulatory functions of Hox transcription factors that can be substituted by other paralogous homologs (Lacombe et al., 2013). Compared to a single mutation, severe phenotypic defects occur only when several paralogous genes or the entire paralogous group are mutated (McIntyre et al., 2007; Wellik et al., 2002; Wellik and Capecchi, 2003). Our research results also demonstrated that most Hox proteins can bind to the predicted target sites upstream of Gtl2. Although the purpose and mechanism of the functional redundancy of Hox proteins are challenging to explain, from the perspective of the role of *Hox* genes in the development of keratinocytes and corneal epithelial cells, the functional redundancy of Hox proteins ensures effective downstream regulation, averting developmental shifts arising from the loss of function of a single protein.

In conclusion, the current investigation elucidates the potential mechanisms by which the Hox/Gtl2-Dio3 miRNA axis influences the determination of identity in keratinocytes and corneal epithelial cells. Hox proteins, acting as transcription factors, are responsible for the maintenance of high expression levels of Gtl2-Dio3 miRNAs in keratinocytes through direct binding to the Gtl2 locus, thereby specifying the keratinocyte identity. Conversely, the silencing of *Hox* genes results in a reduction in the expression of Gtl2-Dio3 miRNAs, which in turn releases transcription factors, including Pax6 and Otx2, as well as signaling pathways. This alteration creates a permissive environment conducive to the development of corneal epithelial cells (Figure 9). These findings offer a plausible avenue for exploring the etiology of corneal opacity and the potential for transdifferentiating keratinocytes into corneal epithelial cells.

## Data Availability

All the raw data generated in this study have been deposited in the NCBI, the accession number is PRJNA1197694.
