# Supplementary material for: Network analysis reveals potential mechanisms that determine the cellular identity of keratinocytes and corneal epithelial cells through the Hox/Gtl2-Dio3 miRNA axis
Source: Front Cell Dev Biol. 2025 Jan 17;13:1475334. doi: 10.3389/fcell.2025.1475334 (PMC11782130; doi:10.3389/fcell.2025.1475334)
Supplement: Supplementary file 2 [file DataSheet5.docx]

**Supplementary Materials and Methods, Figures and Tables**

**Supplementary Materials and Methods**

**Immunofluorescence staining**

For Immunofluorescence staining, cells were fixed with 4% paraformaldehyde for 20 minutes, then permeabilized twice with 0.3% Triton X-100 in PBS for 5 minutes, and blocked in a PBS solution containing 5% bovine serum albumin (BSA) and 0.3% Triton X-100. The primary antibodies diluted in 1% BSA were added, and the cells were incubated overnight at 4°C. After washing three times with PBS, the secondary antibodies were added and incubated at 37°C in the dark for 30 minutes. The cell nuclei were stained with DAPI and observed under a fluorescence microscope. The antibodies were used as follow: rabbit anti-Krt5 monoclonal antibody (ab52635, Abcam) as the primary antibody for keratinocytes; rabbit anti-Krt12 monoclonal antibody (ab185627, Abcam) as the primary antibody for corneal epithelial cells; AlexaFluor-488-conjugated anti-rabbit immunoglobulin G (IgG) (ab150077, Abcam) as the secondary antibody.

**Cell viability assay**

KCs and CECs were seeded in 96-well plates with a concentration of 3×103 cells per well and incubated at 37℃ for 24 hours. Cell viability was assessed at different time points (24h, 36h, 48h, 60h and 72h), using the CCK-8 assay (Beyotime, Shanghai, China) according to the manufacturer’s protocol. The absorbance at 450nm was measured with a microplate reader (Synergy H1, BioTek, Agilent, CA, USA).

**Supplementary Figures**


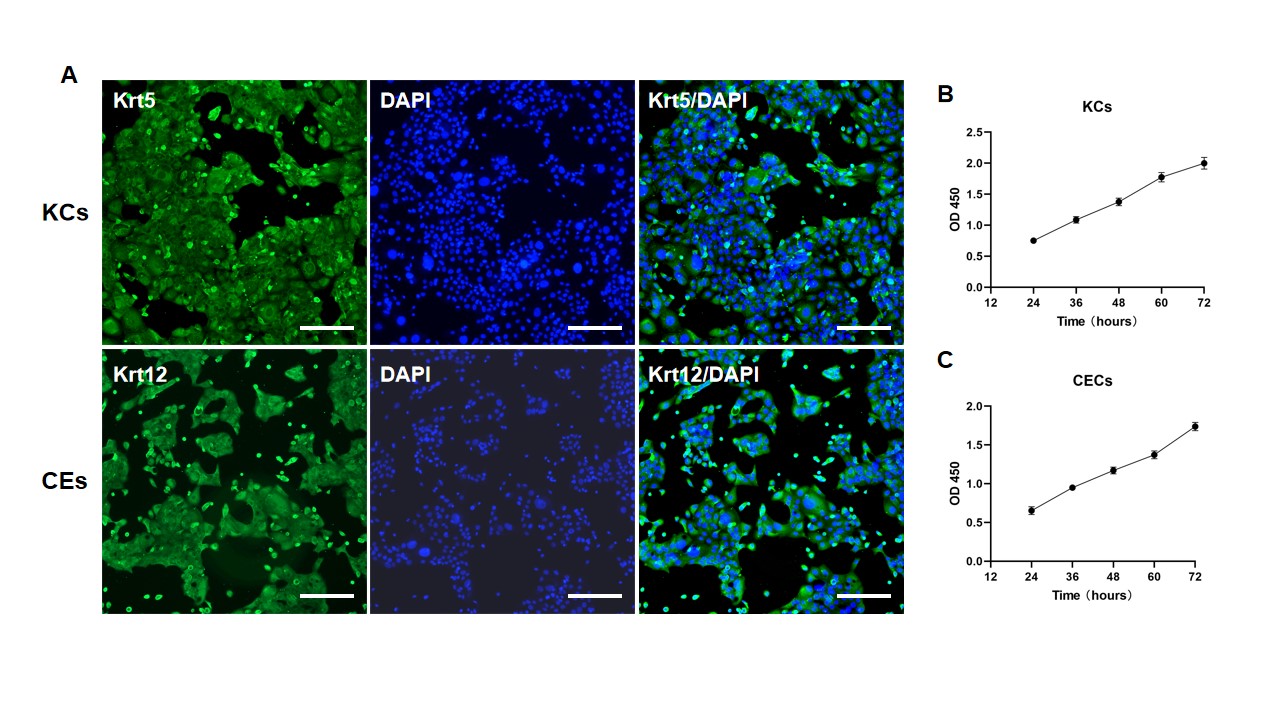


Supplementary Figure 1. Characterization of keratinocytes (KCs) and corneal epithelial cells (CECs).

1. Immunofluorescence staining in KCs and CECs. (B) Cell viability of KCs was determined by CCK-8 assay. (C) Cell viability of CECs was determined by CCK-8 assay.

**Supplementary Tables**

**Supplementary Table 1.** Overall statistics based on the reads of the miRNA sequencing data.

|  | KCs-1 | KCs-2 | KCs-3 | CECs-1 | CECs-2 | CECs-3 |
| --- | --- | --- | --- | --- | --- | --- |
| Raw reads | 22,968,458 | 32,387,852 | 50,640,097 | 16,557,511 | 20,986,177 | 22,983,984 |
| Clean reads | 22,253,266 | 31,037,066 | 48,408,126 | 16,154,164 | 20,476,936 | 22,296,603 |
| Unique reads | 20,526,054 | 28,831,453 | 44,261,484 | 15,264,509 | 19,202,175 | 20,811,288 |
| Mapped reads | 12,207,799 | 20,892,142 | 31,528,349 | 10,980,921 | 14,111,738 | 15,433,961 |
| Known miRNAs | 946 | 993 | 1103 | 861 | 931 | 935 |
| Novel miRNAs | 414 | 444 | 509 | 398 | 459 | 464 |

**Supplementary Table 2.** Overall statistics based on the reads of the mRNA sequencing data.

|  | KCs-1 | KCs-2 | KCs-3 | CECs-1 | CECs-2 | CECs-3 |
| --- | --- | --- | --- | --- | --- | --- |
| Clean Reads | 122,706,402 | 108,227,948 | 110,572,316 | 117,882,326 | 141,197,548 | 109,353,032 |
| Mapped Reads | 103,684,125 | 95,513,295 | 99,464,466 | 109,233,003 | 122,598,875 | 101,482,026 |
| Uniq Map Reads | 99,100,599 | 91,611,148 | 95,355,282 | 104,498,313 | 117,244,825 | 96,982,493 |
| Q30 (%) | 93.76 | 94.27 | 94.12 | 94.47 | 94.60 | 94.54 |
| GC (%) | 54.94 | 51.48 | 51.47 | 51.05 | 54.99 | 50.66 |
