# Supplementary material for: Network analysis reveals potential mechanisms that determine the cellular identity of keratinocytes and corneal epithelial cells through the Hox/Gtl2-Dio3 miRNA axis
Source: Front Cell Dev Biol. 2025 Jan 17;13:1475334. doi: 10.3389/fcell.2025.1475334 (PMC11782130; doi:10.3389/fcell.2025.1475334)
Supplement: Supplementary file 4 [file DataSheet1.docx]

Meg3 Genomic Sequence

>mm10_knownGene_ENSMUST00000143836.7 range=chr12:109539005-109541004 5'pad=0 3'pad=0 strand=+ repeatMasking=none

GCCCTCCTTCTCTCAAGGACACAGGGCTTGGAGCCCAGAGAATGGCTGTTGGGTGTCTGAGGGTCCTGTCAGTGCTTGCCAGAGGCTCACACCAGGCTCCAGCTCACGTTCTTTTGGGGGCTGCAATGCTGACAGCCTTCAGGGACCCCTAGTACATGGCTACTGATGCTCTCTAGTGGCCGCTGCAATCCCACTCGTCGTGGGCCACGAACATGAGCCTCCATGACAATGCGCACCCCTTGCCTCTGCCCCCAGAGGCTAGCAACTGGGCCTTCTGGTGTCTGATTGGCCACAGGGGCTTGACGACAGGGTCTCCTTGCTGATGATTGGCCCCAGCCAGCTCTGGCTGTTTCCTAGCTAATAATACTGTGGGTTAATAAATGTCTCCTGTTGACTATGGTGCTGGGAGCCATGTTTTTTGCTGCATAATGACGTCTGTGGCTGGAGAAGGCCTCTTTAGCTTGTCAGGGGCCTTCTTTTGGGGGGCTCTTTGCAAAAATGGGGTGTTTTCTTTCTCTCCCAGTATCTGACACATTCAGAAAGCTCTTTTTGATGCCCCCAAAGCCCTAAACCTTCATTTTCACCCTCCGAAGTGCATCGGTTCCTACCTTAACAACGCCCCCACCCCTGAAAAGGCCTGAGTCACCTCGCCGGCAAGCTGCTGATCGGTAACCCTGGGCTCAGCGGTGGCTTTTCTCAGTGCCTCTCAGCCCCAGGACCCGAGTCACAGTGGCATCCTAGGCTTGCTGGCCATAGGGGGCTTACGCGAAAGCAGCTGCAGAGGCGTAAATAAGGCGGCGGCGGGGGGGTGGGGGTGGGGGGCTCTTCCAGCTTCATGTCCTCCACAGGGCCTCTGACTTTCCTTTTAGGGTCTCAATCATTCTCGCTCCTTTTCTGTCACCAGACGCTTCTTCTTTGGACTCACAGTTGCCACCCACCTGTCACCCCAAGATAGATCCTTCGTCCCTTTCAACAGCATTCTGACCTTTTCGAGAGGACATTTGATGGACATGTTGCCCCCTTTGCCACTTAGGGTAGGCAGAGCAGCCGGAGGTACCACCTGTTAAGGATTAGCTCTCCTGTGCCAAGTCTCAGAGCGTCCGCAGCCAGCAGCTACGCACCATAGCAACATGTGTCTGCTTGGGCTGCGGAGAGGGGGGGGCGCCCTTCAAAGTGTGGGGAATCAGCCCGATTTGGGGGTGTACTCTAAGCATTACCACAGGGACCCCATTTTCACTAATTAAGTACTTTTCTTAGGGGGCACAGTTGCGCCTATATTCACAGTACACTCCGGGTTGCTGGGTACCCCTGTAACGGGCAGAAATGGGTCCTTGGGAAAGGGCGGAATAGCGCAAGGTTTTTGGTGCACAGGTTGTATCTTCTGTAAAGCCTGGGACTCAAAATCAAGGTCCTTTTGCCTCAACAATGCCAAATTCCCCGTATCAAGATAGTCCGTCAGAATCGGGGTACCCTATGTGGGGGTGACAGCCTCCAGGCTAACATTTGGGAATCAATTATTTTATCTGGGATTTTTATTAAAAATTTCCAGTGCAATTAGGAAAAAACAACGCTCTCCTTTCCTAAGCCGGAGCCCCTGACTGATGTTCTGAGAAACCCAGGCAAGCCATCTGCCGATCCCCGGTACCCCACCTTTATCCTTGGTCGCCTGAGAACAATCACCAGTTGGGGTTATTTCCCCCCAGTTTCTGTCTACAAATCGCCCTTCCATCCACAACTAGGGCTCATGTAGGGAAAAATCACCAGCGACCACAGGGTGTTGGTCATGGCGGCCAGGGGCACTGCGGCAGATTTTTTTTTCCTTCGTTCTTTGCTGCAGTCTGGGTGCGGCTACAGCAATTTGTCATAGAATCTGGGGGGCTCATTTTTCCGGCCAATCACTTTTAGAGAAATGAGCGCATTGCAGCAGAATGCGCTGACGTCAAAGACCACCCCTTCTGCGCCTTTATATAAACCCCACCCAGCCAGCCCCTAGCACAGAAGAATCTCTTACCTGGTGAGTGGTTAGCCATCCTTTGCCTGAAAGGATGTGCAAAAATGAAGACGACATCACTATCTGGCTTCGGCTCCGTCCTCCTGGACATGCCGAAAGGCCAGTGCTGGGGACCTTCTCCCAAAGCCAGCCCCTTAGCCTGGTCCCCAGCATCCAACACGAAATTCTGC

>hg38_knownGene_ENST00000648456.1 range=chr14:100824098-100826097 5'pad=0 3'pad=0 strand=+ repeatMasking=none

TccttctaggggctccaggagcatcccacctgaggcctggggctcccaccggaacgtcaactcatgcccttgtggggctgcagggctgacgcgggctggcactgtgtctacgacagcctcccgggcccccgggtgcgtggctgcggatgctctctggcggccaccacagttcccacgcgcggcgggtgaatgcgagccccttgtcaatgcgtgcccctcgcctctgcccccgagaggctagcaaccgggcctgccggcgtctgattggccgtgggggcctgacgacaggggctcctggttgctgattggccccggccagctctggctgtttcctagctattaatactgtggctaataaacgttctcctgttgacgcgggtgctcggagccatgattttttgaagcgttcttgacgtctgtggctggtgcgggcgtttctgctgtgcaaggcctgcttccggggctctagtccctggggctcctggcaagctccacaggctgtaaagggggtgttttctttctccttcgtgttacctgacatattaaagcagcgccccatgaggaccccaacgtcccacgtctctatctccccaacagtgcgcctgtttatgaaaaaacgagccccccacacgccgtcccaaggctcggcgcctctagtgacctgacggtcaatgttcacctccctagtcattagctgtggacgtagaaatagcctttctcccttcttttcagccctggaatctcccgtctgctttacagtcaccttgtgggatcgttgggcatggggggctccctctgacgccgcttaaaccccccaaagaagtgcgggggagcgattctgacttgatgccctcggcggggcctctctgctgtcctctttggggccccttgctcatcctcacctgctttctgtcaccgagcactccatccttgtcctcctggctgccacccacctgtcaccctaagatagatcctcggtccctttcaacagcattttgacctttgcgagaggacatttgattgacggccctgcacgctctgccactcagagccgggcagagctgccgagggctcccacctgttagggattaactcccatgtgccagctccggagccgaggccgcggcagggctcggcgcaacatgtgtcgctgcctgtttttcggggcgctggggctctttcaaagggcatgtgtgtggggatcaccccaactttgaggggtagctcaggcatctccacatgggacacagtccgccctctaaaagtactttccttagagggcacggctcctgtctggattcttattacaaccctggtgtgtgtggtggggggtacccctggttaaggtggaggaagcggacccagtggaagaggaaggggcatagcgggttccgcgaacatgagttgtaagcggcagagcccgggactccacagccagggttttctgactcggagacgcggagctctgtctcccatgtcaaagagaaccagtcagaaacgcacgggtgggctgggagggggtgacagcctctggcttacatctgggaaccagttatgtcgcccgggcattttattttcctgaacaataagagaaagcatgattttctttcgctaagcccgcatcctccgatggatgttccgaaaccgccaggtgtgggatctgcgccccgacagccccaccttggccatcggccgcctgaggacggccagccatcggggcgattccccccacacattgtgcctgaattcaccctgcctggcgagccgggctcacgcagggaaaaagcacccgcgaccacagggtgttggtcatggcggccaggggcactgcggcagaattttttcctcccttctttgctgcaatctgggtgcggctagagcaatttgtcatagaatctggggggctcatttttccggccaatcacttttagagaaatgagcgcattgcagcagaatgcgctgacgtcagagaccaccccttctgcgcctccatataaaccCCACCCAGCCAGCCCCTAGCGCAGACGGCGGAGAGCAGAGAGGGAGCGCGCCTTGGCTCGCTGGCCTTGGCGGCGGCTCCTCAGGAGAGCTGGGGCGCCCACGAGAGGATCCCTCACCCGgtgagtggttggccatccttgccgcaaaggatgtgcaaaaggaagacggcatccgcttctgggatgggctctgtcctcctggacatgccgagagcctgcctgatcctgggtcctgctgctggaggcggccacttcgcctggtccccgagcgtccgcaacaaaatttgtcagaaagaaaat
